# Wnt4, Wnt6 and β-catenin expression in human placental tissue – is there a link with first trimester miscarriage? Results from a pilot study

**DOI:** 10.1186/s12958-022-00923-4

**Published:** 2022-03-17

**Authors:** Elpiniki Chronopoulou, Vasiliki Koika, Konstantinos Tsiveriotis, Konstantinos Stefanidis, Sotirios Kalogeropoulos, Neoklis Georgopoulos, George Adonakis, Apostolos Kaponis

**Affiliations:** 1 Department of Obstetrics and Gynaecology, University General Hospital of Patras, 265 04 Rion, Greece; 2 Department of Obstetrics and Gynaecology, Division of Reproductive Endocrinology, University General Hospital of Patras, 265 04 Rion, Greece; 3 Department of Obstetrics and Gynaecology, University Hospital of Athens, “Alexandra”, Lourou 4-2, 115 28 Athens, Greece

**Keywords:** β-catenin, Early pregnancy, Miscarriage, Placentation, Wnt

## Abstract

**Background:**

Demystifying the events around early pregnancy is challenging. A wide network of mediators and signaling cascades orchestrate the processes of implantation and trophoblast proliferation. Dysregulation of these pathways could be implicated in early pregnancy loss. There is accumulating evidence around the role of Wnt pathway in implantation and early pregnancy. The purpose of this study was to explore alterations in the expression of Wnt4, Wnt6 and β-catenin in placental tissue obtained from human first trimester euploid miscarriages versus normally developing early pregnancies.

**Methods:**

The study group consisted of first trimester miscarriages (early embryonic demises and incomplete miscarriages) and the control group of social terminations of pregnancy (TOPs). The placental mRNA expression of Wnt4, Wnt6 and β-catenin was studied using reverse transcription PCR and real time PCR. Only euploid conceptions were included in the analysis.

**Results:**

Wnt4 expression was significantly increased in placental tissue from first trimester miscarriages versus controls (*p* = 0.003). No significant difference was documented in the expression of Wnt6 (*p* = 0.286) and β-catenin (*p* = 0.793). There was a 5.1fold increase in Wnt4 expression for early embryonic demises versus TOPs and a 7.6fold increase for incomplete miscarriages versus TOPs – no significant difference between the two subgroups of miscarriage (*p* = 0.533).

**Conclusions:**

This is, to our knowledge, the first study demonstrating significant alteration of Wnt4 expression in human placental tissue, from failed early pregnancies compared to normal controls. Undoubtedly, a more profound study is needed to confirm these preliminary findings and explore Wnt mediators as potential targets for strategies to predict and prevent miscarriage.

## Background

Sporadic miscarriage is a very common complication of early pregnancy and is mostly attributed to high prevalence of chromosomal abnormalities in human embryos [1]. The reported incidence is 31% [2] and it is estimated that 43% of women will go through a miscarriage in their lifetime [3]. It is difficult to estimate the true incidence of first trimester miscarriage as often pregnancies fail early before they are suspected or confirmed. Miscarriage acts often as a natural selection mechanism for abnormal conceptions and this has been described in the literature since the 1980s [4]. However, miscarriages of chromosomically normal conceptions occur, both in spontaneous pregnancies and in assisted reproduction.

Implantation and proper trophoblast proliferation and invasion are amongst the most crucial steps for the establishment and successful progress of the pregnancy [5]. However, the events around early pregnancy are yet to be demystified. These processes are orchestrated through a cascade of molecular events and dysregulation of these pathways could be associated with early pregnancy loss. In the current literature there is accumulating evidence regarding the role of Wnt pathway in early pregnancy [6, 7]. Wnt signaling has been associated both with endometrial receptivity and with embryo implantation potential [8–10]. Alterations in this cascade have also been linked to infertility, recurrent miscarriage (RM), endometriosis, endometrial cancer, gestational trophoblastic disease, pre-eclampsia and fetal growth restriction [6].

### Wnt family

Wnt family of glycoproteins act as intercellular signals to regulate cell proliferation and are highly conserved in the evolution of species. They were discovered in 1982 and consist of 19 proteins of 12 subfamilies [11–13]. They act as signaling molecules and activators of transcription factors. Wnt signaling consists of a canonical pathway which acts through β-catenin and a more complex but less studied, β-catenin independent, non-canonical pathway [14]. There are also other, less studied branches, with distinct signaling effects [15].

In a simplified description of the canonical pathway, in the absence of Wnt activity, β-catenin is phosphorylated by a “destruction complex” which results in proteasome degradation. Thus, low levels of β-catenin enter the nucleus and the expression of target genes is inhibited. The presence of Wnt ligands inhibits the destruction of β-catenin after binding to the frizzled receptor (Fz) and its co-receptor lipoprotein receptor-related protein (LRP) 5/6. As a result, β-catenin accumulates, is transferred to the nucleus and regulates gene transcription by activating transcription factors such as T cell factor/lymphoid enhancer binding factor (TCF/LEF) [16].

The non-canonical pathway consists of the Wnt/Ca2 + and planar cell polarity (PCP) components and has a big network of receptors and co-receptors, whose combinations and availability in specific tissues and contexts determine the downstream effect [15]. This pathway is tangled with other cascades and possibly interferes with the canonical pathway either inducing β-catenin destruction or favoring canonical pathway inhibitors such as Dkk-1 (dickkopf-related protein 1) [17, 18]. The dickkopf family of Wnt pathway antagonists acts through binding to LRP receptors causing their endocytocis. Their expression in human endometrium is regulated by progesterone [19]. Other antagonists [secreted frizzled related proteins (sFRPs), Wnt inhibitory factor 1 (WIF1) and Cerberus] bind directly on Wnt proteins [20].

### Wnt pathway and implantation

There is accumulating evidence, mostly derived from animal models, which suggests that Wnt signaling is key for placental development and is implicated in the regulation of trophoblast proliferation and invasion [21–24]. Targeted disruption of the expression of Wnt components has been associated with a large spectrum of gestational diseases in mice. Wnt7b null mice died midgestation due to placental abnormalities [21]. Similarly, half mice with Wnt2 disruption died perinatally with alterations in both the size and structure of placentas [22]. Wnt/β-catenin silencing has been shown to block the ability of the blastocyst to implant [25]. Interestingly, Bao et al. (2020) recently showed that over-expression of the Wnt-β-catenin signaling, through silencing of Wnt inhibitors, is also detrimental for trophoblast differentiation [26].

In human pregnancies the role of the Wnt pathway in placentation is poorly understood [27]. Authors have mostly used choriocarcinoma model systems and immortalized trophoblast cell lines. Fourteen Wnt ligands and eight Fz receptors have been identified in human first trimester placental tissue [28]. Amongst them Wnt4 is one of the most abundantly expressed while Wnt6 is lowly expressed. Epigenetic studies have shown promoter methylation for four Wnt inhibitors in human term placentas and in trophoblast tissue [29]. The Wnt pathway seems to be closely associated with the invasive differentiation process of human trophoblast and with the cross-talking between the trophoblast and the decidua. Treatment of decidualized human endometrial stromal cells with conditioned media from human trophoblast resulted in the downregulation of Fzd and Wnt4 [30]. Wnt-Dependent TCF4 has been shown to regulate human trophoblast differentiation and motility in trophoblast cell models [31].Loss of function of β-catenin in human embryos affected their ability to blastulate and resulted in significantly fewer trophectoderm cells. Exposure of embryos to Wnt3, in the same series of experiments, promoted the progenitor trophoblast lineage differentiation [32]. Furthermore, vascularization of primary villi seems to be Wnt -dependent [33] as the Wnt signaling is implicated in the regulation of angiogenic factors such as VEGF (vascular endothelial growth factor) [34].

Similarly to the animal models, balanced action of the Wnt signaling is important for human placentation. Both hyper-activation and under-activation of Wnt signaling has been associated with placenta-related pathologies and trophoblast disorders. The downregulation of canonical pathway inhibitors, such as Dkk, negatively affects trophoblast invasion [35]. Hyperactivation of the canonical cascade is linked to choriocarcinoma [36] and molar pregnancy [37]. Increased Dkk-1 and sFRP4 expression, decreased Wnt2, Wnt4 and β-catenin expression, increased and decreased Wnt5a expression, have all been associated by different authors with the development of pre-eclampsia [38–41].

As discussed above, there is evidence, mostly from animal studies, that Wnt4 and β-catenin have a role to play in implantation and normal placental development. The literature exploring the expression of Wnt6 during implantation and decidualization is, however, limited. The study by Wang et al. (2013) was the first to demonstrate that Wnt6 is also critical for normal stromal cell proliferation during decidualization in mice [42]. Zhang et al. (2015) documented four Wnt6 mutations in the coding sequence of Wnt6 in women with unexplained RM [43].

The aim of this study is to look into the expression of Wnt4, Wnt6 and β-catenin in human placental tissue obtained from first trimester miscarriages versus normally developing early pregnancies, in order to detect a possible link between alterations in this signaling pathway and early pregnancy failure in humans.

## Methods

### Patient recruitment

Patients were recruited at University Hospital of Patras, Department of Obstetrics and Gynaecology from July 2017 to August 2018. All women included in the study signed informed consent and were fully informed regarding the research protocol and study question. The research protocol adheres to the guidelines in Declaration of Helsinki and is approved by the Medical School of the University of Patras, the Ethics Research Committee and the Scientific Board of University Hospital of Patras.

The study group consisted of first trimester miscarriages. The control group consisted of healthy women, with at least one previous live birth and no history of miscarriage or stillbirth, who attended for elective termination of pregnancy (TOP) for social reasons.

The diagnosis of first trimester miscarriage was defined according to criteria formed by the Society of Radiologists in Ultrasound (Multispecialty Panel on Early First Trimester Diagnosis of Miscarriage and Exclusion of a Viable Intrauterine Pregnancy) as published in 2013 [44]. Early embryonic demise was defined with a transvaginal ultrasound scan as crown-rump length of 7 mm or more and no detected heartbeat or mean gestational sac diameter 25 mm or more and no visible fetal pole. A second opinion was sought for the viability of the pregnancy to confirm the diagnosis. For all cases of incomplete miscarriage, previous scan had confirmed an intrauterine pregnancy and two users confirmed the presence of products of conception on transvaginal ultrasound examination. For the control group, before surgical TOPs for maternal request, the viability and normal progression of pregnancy was confirmed on two occasions at least one week apart by transvaginal ultrasound including immediately before the procedure.

Exclusion criteria for both groups included gestational age more than 12 + 6 weeks, assisted conception, multiple pregnancy, chronic illness, known risk factors for miscarriage such as antiphospholipid syndrome or uterine anomalies, history of RM and use of any hormonal medications such as progesterone.

### Surgical management and tissue sample collection

For both miscarriages and elective terminations surgical evacuation of products of conception was performed under general anesthesia according to the department’s protocol. All patients signed informed consent. All patients had blood sample taken for full blood count and group and save before the procedure. After cleaning and draping in the lithotomy position and examination under anesthesia, the cervix, unless already open, was dilated to Hegar 8–10 under transabdominal ultrasound guidance. A rigid suction curette was used to empty the uterus of products of conception under ultrasound guidance. Tissue was collected in a closed system leading to a sterile container. Uterus was checked empty with gentle 360^0^ curettage. Uterotonics were administered. Good uterine tone and haemostasis were confirmed. Patients were monitored in recovery after the procedure and were discharged the same day. Anti-D immunoglobulin was prescribed for Rhesus negative women. All procedures were uncomplicated. There were no hospital readmissions.

Products of conception were obtained under aseptic conditions. These fresh first trimester tissue samples consisted of blood clots, decidua tissue, villous tissue, complete or fragmented gestational sac and embryonic tissues or an embryo. Gross examination allowed identification of maternal, placental and embryonic components. Chorionic villi and decidua were identified microscopically and separated using forceps and scissors if necessary. Contaminating maternal blood clots were removed [45, 46]. The early placental tissue was washed four times in sterile saline and transferred to medium containing antibiotic–antimycotic mixture.

Biological specimens were preserved in RNAlater buffer until RNA extraction. Only euploid conceptions (confirmed by karyotype) were included in the analysis.

### Real time polymerase reaction analysis

Isolation of total RNA was carried out, including a 15-min DNAse I treatment, using the commercially available RNeasy Lipid Tissue Mini kit provided by QIAGEN according to the manufacturer’s protocol. RNA concentration and purity were estimated by measuring optical absorption at 260 nm and calculating the ratio 260/280 nm, respectively. Complementary DNA (cDNA) synthesis was performed using the Transcriptor First Strand cDNA Synthesis Kit (04,379,012,001; Roche Applied Science) with a mixture of anchored-oligo(dT)18 primer and 1 μg of total RNA according to the manufacturer’s instructions. Genes relative expression was estimated by real-time PCR in the LightCycler 2 Instrument, Roche using FastStart Universal SYBR Green Master (Roche Hellas). The PCR primers for each gene are presented in Table 1 and have been previously published [39, 47–49]. Finally, mRNA levels were normalized to the Alu-Sq levels to account for possible variation in the amount and quality of RNA between different samples. The quality of the PCR reactions was confirmed by melting curve analysis and the 2 − ∆∆Cp algorithm was used to analyze the relative expression of the target genes [50].

**Table 1:** PCR primers for each gene (Wnt4, Wnt6, β-catenin and Alu)

Gene	Forward primer	Reverse primer
Wnt4	CCTTCGTGTACGCCATCTCT	TCAGAGCATCCTGACCACTG
Wnt6	TCCGCCGCTGGAATTG	AGGCCGTCTCCCGAATG
β-catenin	GAGGGGTGGGCTGGTATCTC	CTCGACCAAAAAGGACCAGA
Alu	CATGGTGAAACCCCGTCTCTA	GCCTCAGCCTCCCGAGTAG

Several steps were taken to minimize the possibility of DNA contamination; the RNA extraction protocol included a step of DNase treatment, the primers were designed to hybridize to different exons and a melting curve analysis was performed at the final step of real time PCR.

### Statistical analysis

Statistical analysis was performed using SPSS *(IBM Corp. Released 2017. IBM SPSS Statistics for Windows, Version 25.0. Armonk, NY: IBM Corp.)*. Continuous variables were compared using independent samples t-test and Mann–Whitney U test in case of normally and not-normally distributed data respectively. Normal distribution was assessed using Kolmogorov Smirnov test and Shapiro Wilk tests. Categorical variables were assessed using Fisher’s exact test. Normally distributed variables are reported as mean ± SD while non-normally distributed continuous variables are presented as median (1st quantile, 3rd quantile). Power analysis was performed using NCSS PASS. A two-sided p-value < 0.05 was considered statistically significant.

## Results

The studied samples comprised of 42 placental tissue samples; 28 obtained from first trimester miscarriages (study group) and 14 obtained from elective TOPs (controls). The miscarriage group consisted of two subgroups (14 early embryonic demises and 14 incomplete miscarriages) and statistical analysis was performed to identify differences in expression between miscarriages and TOPs as well as between these two subgroups.

The mean gestational age (mean ± SD) was 8.9 ± 1.4 weeks of pregnancy for the miscarriage group and 8.2 ± 0.7 weeks for the control group (*p* = 0.088). The mean age of the included women at the time of conception was 34.0 ± 6.8 years for the miscarriage group and 32.4 ± 6.4 years for the TOP group (*p* = 0.420). There were also no significant differences in patients’ demographic background such as BMI (body mass index) and smoking status between the study group and TOPs. The demographics are summarized in Table 2. None of the included women in the study group had RMs. The control group consisted of women with at least one previous live birth and no previous miscarriages.

**Table 2:** Characteristics of the study population and control group

	N	Age in years (median, Q1-Q3)	GA in weeks (*n* ± SD)	BMI in kg/m^2^ (*n* ± SD)	Smoking (n)
TOP	14	36.0, 27.3–40.0	8.2 ± 0.7	22.5 ± 2.1	3
Miscarriage	28	32.0, 26.8–38.0	8.9 ± 1.4	21.8 ± 1.9	4
P		0.420 ^*^	0.088 ^†^	0.276 ^†^	0.668 ^‡^

*BMI* body mass index, *GA* gestational age, *SD* standard deviation, *Q1* 1^st^ quantile, *Q3* 3^rd^ quantile, *TOP* termination of pregnancy

^*^: Mann–Whitney U test; †: Independent samples t-test; ‡: Fisher’s exact test

**Fig.1:**
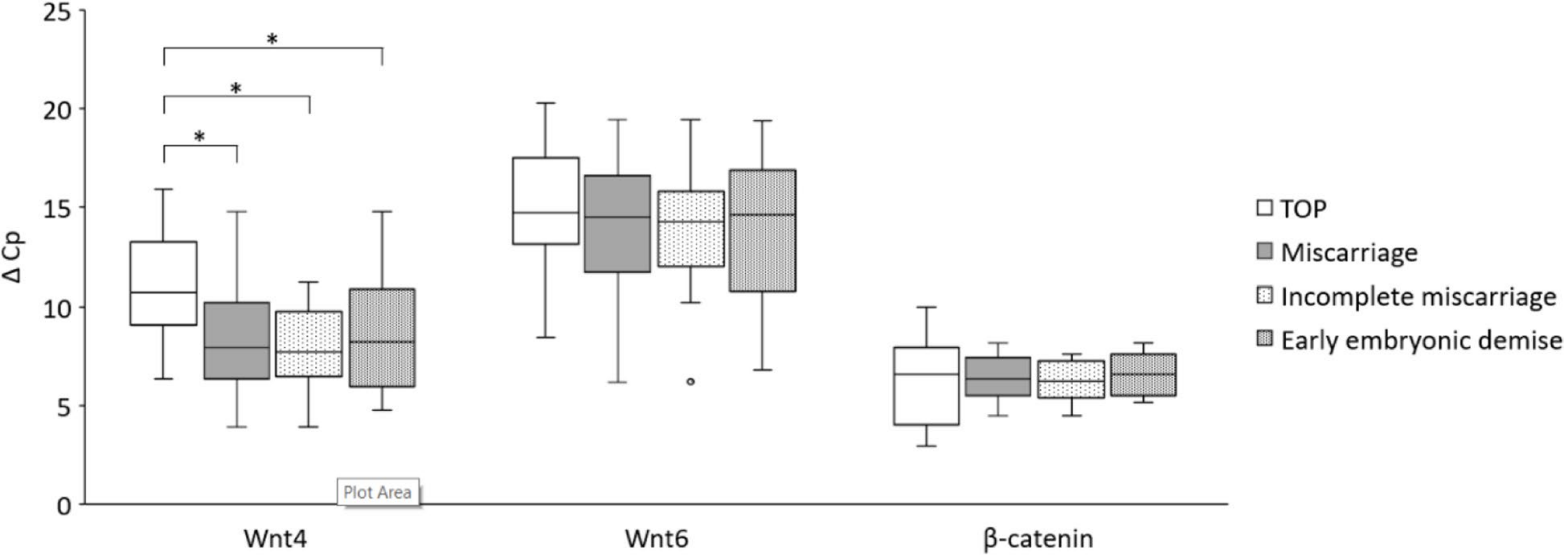
Box plot of ΔCp values of studied genes for each population independent sample t test, * p < 0.05. TOP: termination of pregnancy

**Fig.2:**
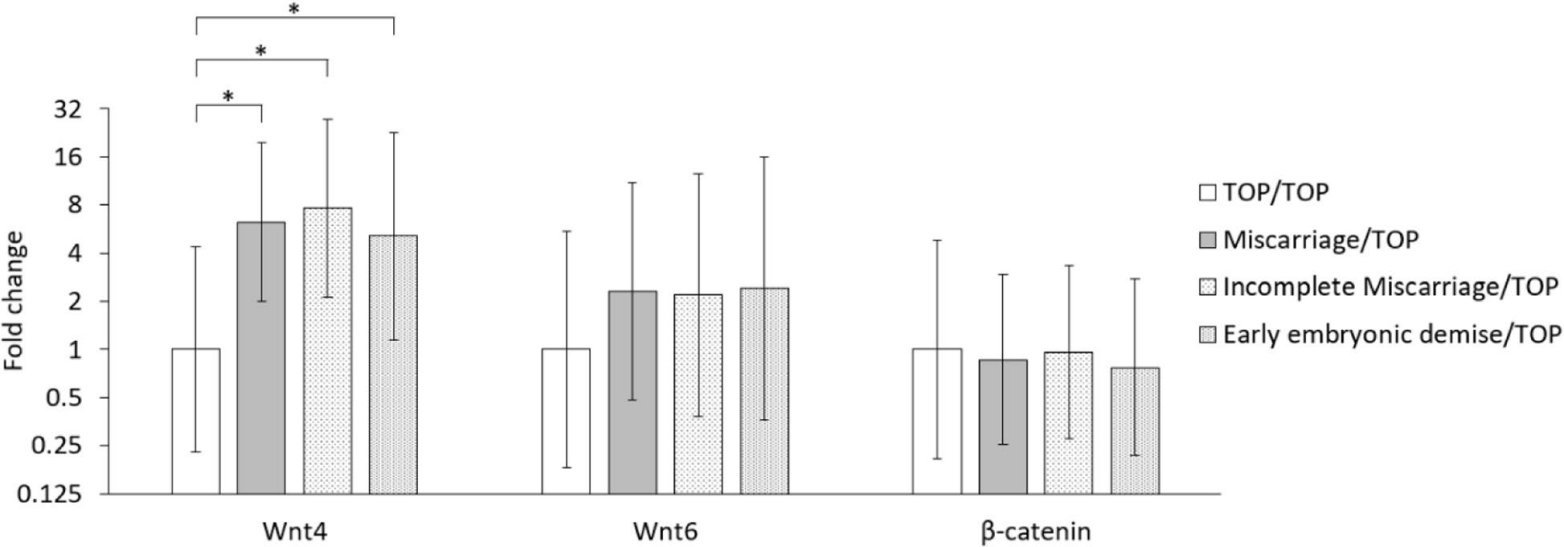
Relative expression of studied genes compared to control group (TOP) 95% confidence interval, independent sample t test, * p < 0.05. TOP: termination of pregnancy

A statistically significant increase of Wnt4 mRNA expression was noted in placental tissue obtained from first trimester miscarriages versus TOPs. No statistically significant difference was documented for the mRNA expression of Wnt6 and β-catenin between miscarriages and TOPs. The results, for each studied gene, are depicted in Figs. 1 and 2 and Table 3.

**Table 3:** ΔCp values of studied genes for each population

	**Wnt4**			**Wnt6**			**β-catenin**		
	n	Mean	SD	n	mean	SD	n	mean	SD
TOP	14	10.8	2.7	14	15.0	3.2	14	6.3	2.2
Miscarriage	28	8.2	2.4	28	13.8	3.5	28	6.4	1.1
Incomplete Miscarriage	14	7.9	2.0	14	13.9	3.3	14	6.3	1.0
Early embryonic demise	14	8.5	2.8	14	13.7	3.8	14	6.6	1.1

*SD* standard deviation, *TOP* termination of pregnancy

There was a significant difference in the ΔCp of Wnt4 between miscarriages and TOPs (*p* = 0.003, power = 0.879). When assessing the subgroups of the study group, a statistically significant difference in Wnt4 mRNA expression was also identified between TOPs and each subgroup—incomplete miscarriages (*p* = 0.003, power = 0.877) and early embryonic demises (*p* = 0.033, power = 0.581) (Fig. 1). There was a 6.2fold increase in the expression of Wnt4 mRNA in miscarriages compared to TOPs. There was a 5.1fold increase in the early embryonic demise sub-group compared to terminations and 7.6fold increase in incomplete miscarriages compared to terminations – this difference between the two sub-groups was not statistically significant (*p* = 0.533) (Fig. 2). There was no statistically significant difference in the expression of Wnt6 mRNA in miscarriages compared to TOPs (*p* = 0.286) nor for the expression of β-catenin between the studied group and the control group (*p* = 0.820) (Fig. 1).

## Discussion

The data we have, so far, for Wnt pathway in placental tissue are mostly derived from animal models. There are interesting in vitro studies designed to include cultures of human endometrial stromal cells (HESCs) co-cultures and trophoblast stem cell models [51, 52]. There are also scarce evidence using human endometrial samples [51], blood samples [43] or placental tissue at delivery [39, 53]. Only few studies have looked at human trophoblast in an effort to elucidate the events around early pregnancy [28, 52, 54]. Two of these studies have looked into unexplained RMs (defined as two or more consecutive pregnancy losses before the 20th week of gestation) versus normal pregnancies [52, 54]. The authors documented significantly reduced mRNA expression of β-catenin and of Wnt2 and increased expression of Dkk-1 (Wnt antagonist) in the RM group compared to normal controls. Therefore, the results point towards an association between reduced expression of the canonical Wnt pathway and recurrent pregnancy loss.

Our study is, to our knowledge, the first study demonstrating significantly increased Wnt4 expression in human trophoblast obtained from failed early pregnancies compared to normal controls, suggesting that increased trophoblast expression of a Wnt component could have detrimental effect on early pregnancy.

### Wnt4

Wnt4 was selected as it is the most well studied component of the Wnt family in the adult uterus. It seems to be key player for implantation and is the most abundant Wnt component in the decidua [55, 56]. Wnt4 ablation in mice resulted in embryos which could attach but not invade and endometrial stroma cells unable to decidualize, characterized by attenuated progesterone signaling [57]. The attachment reaction, crucial for decidualization and implantation is characterized by localized stroma induction of Wnt4 gene in the mouse model [9].

This is the first study to look into this component in human trophoblast and our results document that significant increase in Wnt4 placental expression could also be detrimental for early pregnancy suggesting that balanced Wnt signaling is key for early pregnancy progression. The importance of this fine tuning has also been documented by other authors; co-culture models have revealed that the interaction between trophoblast and decidua results in downregulation of Wnt components (including Wnt4) through paracrine signaling [30, 58, 59] and that downregulation of certain Wnt components may be essential for normal pregnancy progression [60]. A recent study by Bao et al. (2020) [26] documented that hyperactivation of Wnt signaling in the mouse model disrupted trophoblast differentiation. The authors observed that two inhibitors of Wnt signaling (sFRPs1 and 5) are highly expressed in extraembryonic trophoblast. They subsequently utilized double knockout mice for these two inhibitors and they observed impaired trophoblast development. Moreover, hyperactivation of the Wnt pathway, through a stabilized form of β-catenin, led to exhaustion of trophoblast precursor cells leading to impaired placentation.

Even though Wnt4 can activate the canonical pathway in the presence of LRP5/6, it is considered predominately component of the non-canonical pathway and is classified as non-canonical Wnt signaling molecule [39, 61]. This may suggest that increased activation of the non-canonical pathway can be problematic in early pregnancy.

When analyzing the two subgroups of early pregnancy failure (early embryonic demise and incomplete miscarriage), in the incomplete miscarriage group, Wnt4 expression is 1.5fold higher than in the early embryonic demise group. Even though this difference between the two subgroups is not statistically significant, this trend could be another demonstration of a possible role of Wnt4 in embryo attachment and placentation. Such trend, if confirmed in larger studies, could contribute in understanding why an early pregnancy loss can present clinically as incomplete miscarriage (with obvious deranged placental attachment at the time of diagnosis) or could present as an early embryonic demise with intact gestational sac.

### Wnt6

Previous authors have documented Wnt6 implication in decidualization [42] and one research team suggested a possible role of Wnt6 mutations in blood samples from Chinese women suffering RM [43]. However, there are no studies clearly linking this component to placentation. Our finding of not statistically significant alteration of Wnt6 expression in trophoblast obtained after early pregnancy loss suggests that possibly Wnt6 is not significantly implicated in trophoblast function at this stage of pregnancy.

### B-catenin

The evidence around the role of β-catenin in early pregnancy remains controversial. There are several mouse studies linking the attenuation of Wnt/β-catenin (canonical pathway) signaling with early pregnancy failure. Under-expression of this pathway seems to affect the blastocyst implantation potential [25], the decidualization process [62] and cell adhesion causing embryo fragmentation [63]. In humans, reduced β-catenin expression was detected in villi obtained from 20 RM cases compared to normal early pregnancies [52]. However, β-catenin gain of function also seems to be detrimental for early pregnancy in mice leading to impaired implantation and decreased decidualization [26, 62, 64]. The role of β-catenin in early placentation warrants more research. This work did not demonstrate significant difference in the mRNA expression of β-catenin in early placental tissue from miscarriages versus normal controls. However, β-catenin is largely regulated at the protein level as a downstream effect of the complicated canonical pathway and protein expression could be influenced by various factors especially in the context of early pregnancy loss and its surgical management.

### Limitations

The control group of normally developing pregnancies consisted of social TOPs. For this group of patients, fetal heart activity was detected on the day of the procedure and the pregnancy appeared to progress normally on two ultrasound examinations at least a week apart. However, we cannot be certain that these pregnancies would ultimately progress normally. Furthermore, gene expression was explored at the mRNA level. In relevant studies, the real time PCR results were verified with protein expression findings [39, 52]. B-catenin, as a downstream factor of the canonical pathway, is largely regulated at protein level. However, Wnt4 is mainly considered activator of the non-canonical pathway and therefore Wnt4 upregulation would not be expected to affect β-catenin protein expression through non-canonical pathway stimulation [39]. Furthermore, the canonical pathway can be initiated by various Wnt components and the deregulation of a component can cause buffering changes in other stimulators of this complex cascade therefore we would not be able to pinpoint a direct downstream effect on β-catenin protein [42]. Since various Wnt components can exert their action both through the canonical and through β-catenin independent cascades and can also interact with different ligands to provoke different responses in different tissues or time points, the downstream network cannot be easily defined [27].

### Strengths

This is, to our knowledge, the first human study to demonstrate statistically significant alteration of Wnt4 mRNA trophoblast expression in miscarriages versus normally developing pregnancies. Despite the small sample size, this study achieved adequate statistical power. There were no statistically significant differences in demographic characteristics of the included population between the study and control groups. Furthermore, patients who received progesterone supplementation were excluded. This is important as progesterone has been shown to affect Wnt signaling and Wnt4 seems to be key regulator of progestogen signaling in the mouse model [57, 65, 66].

## Conclusions

There is accumulating evidence highlighting the importance of Wnt pathway for early placentation but there is place for more research in the field with greater focus on human pregnancy.

The early human placenta is incredibly complex and heterogeneous [45]. Twenty distinct cell types have been identified. The expression of Wnt ligands may differ depending on the cell type and the stage of pregnancy. An attempt to map this expression would yield valuable information for the Wnt pathway and would also contribute to a deeper understanding around placentation.

Our preliminary findings highlight the significance of balanced Wnt signaling for the carefully orchestrated events around early pregnancy and contribute to the existing literature around the Wnt cascade. Further studies are needed to confirm the observed associations and their clinical relevance. Future research could explore Wnt mediators as potential targets for strategies to predict and prevent miscarriage.

## Data Availability

The datasets generated during and/or analysed during the current study are available from the corresponding author on request.

## Abbreviations

APC: Axin, adenomatous polyposis coli
ΒΜΙ: Body mass index
cDNA: Complementary DNA (deoxyribonucleic acid)
Dkk-1: Dickkopf-related protein 1
Fz: Frizzled receptor
HESCs: Human endometrial stromal cells
LPR: Lipoprotein receptor-related protein
mRNA: Messenger ribonucleic acid
NICE: National Institute for Health and Care Excellence
PCP: Planar cell polarity
PCR: Polymerase chain reaction
RM: Recurrent miscarriage
sFRPs: Secreted FZD-related proteins
TCF/LEF: T cell factor/lymphoid enhancer binding factor
TOP: Termination of pregnancy
VEGF: Vascular endothelial growth factor
WIF1: Wnt inhibitory factor-1
Wnt: Wingless

## References

[1] Macklon NS, Geraedts JP, Fauser BC (2002). Conception to ongoing pregnancy: the 'black box' of early pregnancy loss. Hum Reprod Update.

[2] Magnus MC, Wilcox AJ, Morken NH, Weinberg CR, Haberg SE (2019). Role of maternal age and pregnancy history in risk of miscarriage: prospective register based study. BMJ (Clinical research ed).

[3] Cohain JS, Buxbaum RE, Mankuta D (2017). Spontaneous first trimester miscarriage rates per woman among parous women with 1 or more pregnancies of 24 weeks or more. BMC Pregnancy Childbirth.

[4] Plachot M, de Grouchy J, Junca AM, Mandelbaum J, Turleau C, Couillin P (1987). From oocyte to embryo: a model, deduced from in vitro fertilization, for natural selection against chromosome abnormalities. Ann Genet.

[5] Gupta SK, Malhotra SS, Malik A, Verma S, Chaudhary P (2016). Cell Signaling Pathways Involved During Invasion and Syncytialization of Trophoblast Cells. Am J Reprod Immunol.

[6] Sonderegger S, Pollheimer J, Knofler M (2010). Wnt signalling in implantation, decidualisation and placental differentiation–review. Placenta.

[7] Bao SH, Shuai W, Tong J, Wang L, Chen P, Duan T (2013). Increased Dickkopf-1 expression in patients with unexplained recurrent spontaneous miscarriage. Clin Exp Immunol.

[8] Mohamed OA, Jonnaert M, Labelle-Dumais C, Kuroda K, Clarke HJ, Dufort D (2005). Uterine Wnt/beta-catenin signaling is required for implantation. Proc Natl Acad Sci U S A.

[9] Paria BC, Ma W, Tan J, Raja S, Das SK, Dey SK (2001). Cellular and molecular responses of the uterus to embryo implantation can be elicited by locally applied growth factors. Proc Natl Acad Sci U S A.

[10] Tulac S, Nayak NR, Kao LC, van Waes M, Huang J, Lobo S (2003). Identification, Characterization, and Regulation of the Canonical Wnt Signaling Pathway in Human Endometrium. J Clin Endocrinol Metab.

[11] Nusse R, Varmus HE (1982). Many tumors induced by the mouse mammary tumor virus contain a provirus integrated in the same region of the host genome. Cell.

[12] Clevers H (2006). Wnt/beta-catenin signaling in development and disease. Cell.

[13] Niehrs C (2012). The complex world of WNT receptor signalling. Nat Rev Mol Cell Biol.

[14] Ackers I, Malgor R (2018). Interrelationship of canonical and non-canonical Wnt signalling pathways in chronic metabolic diseases. Diab Vasc Dis Res.

[15] Komiya Y, Habas R (2008). Wnt signal transduction pathways. Organogenesis.

[16] Clevers H, Nusse R (2012). Wnt/beta-catenin signaling and disease. Cell.

[17] Topol L, Jiang X, Choi H, Garrett-Beal L, Carolan PJ, Yang Y (2003). Wnt-5a inhibits the canonical Wnt pathway by promoting GSK-3-independent beta-catenin degradation. J Cell Biol.

[18] Park HW, Kim YC, Yu B, Moroishi T, Mo JS, Plouffe SW (2015). Alternative Wnt Signaling Activates YAP/TAZ. Cell.

[19] Tulac S, Overgaard MT, Hamilton AE, Jumbe NL, Suchanek E, Giudice LC (2006). Dickkopf-1, an Inhibitor of Wnt Signaling, Is Regulated by Progesterone in Human Endometrial Stromal Cells. J Clin Endocrinol Metab.

[20] Kawano Y, Kypta R (2003). Secreted antagonists of the Wnt signalling pathway. J Cell Sci.

[21] Parr BA, Cornish VA, Cybulsky MI, McMahon AP (2001). Wnt7b regulates placental development in mice. Dev Biol.

[22] Monkley SJ, Delaney SJ, Pennisi DJ, Christiansen JH, Wainwright BJ (1996). Targeted disruption of the Wnt2 gene results in placentation defects. Development.

[23] Ishikawa T, Tamai Y, Zorn AM, Yoshida H, Seldin MF, Nishikawa S (2001). Mouse Wnt receptor gene Fzd5 is essential for yolk sac and placental angiogenesis. Development.

[24] Lu J, Zhang S, Nakano H, Simmons DG, Wang S, Kong S (2013). A positive feedback loop involving Gcm1 and Fzd5 directs chorionic branching morphogenesis in the placenta. PLoS Biol.

[25] Xie H, Tranguch S, Jia X, Zhang H, Das SK, Dey SK (2008). Inactivation of nuclear Wnt-beta-catenin signaling limits blastocyst competency for implantation. Development (Cambridge, England).

[26] Bao H, Liu D, Xu Y, Sun Y, Mu C, Yu Y (2020). Hyperactivated Wnt-β-catenin signaling in the absence of sFRP1 and sFRP5 disrupts trophoblast differentiation through repression of Ascl2. BMC Biol.

[27] Knofler M, Pollheimer J (2013). Human placental trophoblast invasion and differentiation: a particular focus on Wnt signaling. Front Genet.

[28] Sonderegger S, Husslein H, Leisser C, Knofler M (2007). Complex expression pattern of Wnt ligands and frizzled receptors in human placenta and its trophoblast subtypes. Placenta.

[29] Novakovic B, Rakyan V, Ng HK, Manuelpillai U, Dewi C, Wong NC (2008). Specific tumour-associated methylation in normal human term placenta and first-trimester cytotrophoblasts. Mol Hum Reprod.

[30] Hess AP, Hamilton AE, Talbi S, Dosiou C, Nyegaard M, Nayak N (2007). Decidual stromal cell response to paracrine signals from the trophoblast: amplification of immune and angiogenic modulators. Biol Reprod.

[31] Meinhardt G, Haider S, Haslinger P, Proestling K, Fiala C, Pollheimer J (2014). Wnt-Dependent T-Cell Factor-4 Controls Human Etravillous Trophoblast Motility. Endocrinology.

[32] Krivega M, Essahib W, Van de Velde H (2015). WNT3 and membrane-associated β-catenin regulate trophectoderm lineage differentiation in human blastocysts. Mol Hum Reprod.

[33] Herr F, Horndasch M, Howe D, Baal N, Goyal P, Fischer S (2014). Human placenta-derived Wnt-5a induces the expression of ICAM-1 and VCAM-1 in CD133(+)CD34(+)-hematopoietic progenitor cells. Reprod Biol.

[34] Newman AC, Hughes CCW (2012). Macrophages and angiogenesis: a role for Wnt signaling. Vascular Cell.

[35] Peng S, Li J, Miao C, Jia L, Hu Z, Zhao P (2008). Dickkopf-1 secreted by decidual cells promotes trophoblast cell invasion during murine placentation. Reproduction.

[36] Zeng X, Zhang Y, Xu H, Zhang T, Xue Y, An R (2018). Secreted Frizzled Related Protein 2 Modulates Epithelial-Mesenchymal Transition and Stemness via Wnt/β-Catenin Signaling in Choriocarcinoma. Cell Physiol Biochem.

[37] Pollheimer J, Loregger T, Sonderegger S, Saleh L, Bauer S, Bilban M (2006). Activation of the canonical wingless/T-cell factor signaling pathway promotes invasive differentiation of human trophoblast. Am J Pathol.

[38] Chen Y, Zhang Y, Deng Q, Shan N, Peng W, Luo X (2016). Wnt5a inhibited human trophoblast cell line HTR8/SVneo invasion: implications for early placentation and preeclampsia. J Matern Fetal Neonatal Med.

[39] Wang G, Zhang Z, Chen C, Zhang Y, Zhang C (2016). Dysfunction of WNT4/WNT5A in deciduas: possible relevance to the pathogenesis of preeclampsia. J Hypertens.

[40] Zhang Z, Zhang L, Zhang L, Jia L, Wang P, Gao Y (2013). Association of Wnt2 and sFRP4 expression in the third trimester placenta in women with severe preeclampsia. Reprod Sci.

[41] Zhang Z, Li H, Zhang L, Jia L, Wang P (2013). Differential expression of β-catenin and Dickkopf-1 in the third trimester placentas from normal and preeclamptic pregnancies: a comparative study. Reprod Biol Endocrinol.

[42] Wang Q, Lu J, Zhang S, Wang S, Wang W, Wang B et al. Wnt6 Is Essential for Stromal Cell Proliferation During Decidualization in Mice1. Biol Reprod. 2013;88:5, 1–9–5, 1–9;doi:10.1095/biolreprod.112.104687 10.1095/biolreprod.112.104687 23175771

[43] Zhang Y, Li G, Fan Y, Cui Y, Huang S, Ma J (2015). Novel missense mutation in WNT6 in 100 couples with unexplained recurrent miscarriage. Hum Reprod.

[44] Doubilet PM, Benson CB, Bourne T, Blaivas M, Barnhart KT, Benacerraf BR (2013). Diagnostic criteria for nonviable pregnancy early in the first trimester. N Engl J Med.

[45] Suryawanshi H, Morozov P, Straus A, Sahasrabudhe N, Max KEA, Garzia A et al. A single-cell survey of the human first-trimester placenta and decidua. Sci Adv. 2018;4:eaau4788-eaau;doi:10.1126/sciadv.aau4788 10.1126/sciadv.aau4788 PMC6209386 30402542

[46] Li S, Li N, Zhu P, Wang Y, Tian Y, Wang X (2015). Decreased beta-catenin expression in first-trimester villi and decidua of patients with recurrent spontaneous abortion. J Obstet Gynaecol Res.

[47] Vandesompele J, De Preter K, Pattyn F, Poppe B, Van Roy N, De Paepe A (2002). Accurate normalization of real-time quantitative RT-PCR data by geometric averaging of multiple internal control genes. Genome Biology.

[48] Lu D, Zhao Y, Tawatao R, Cottam HB, Sen M, Leoni LM (2004). Activation of the Wnt signaling pathway in chronic lymphocytic leukemia. Proc Natl Acad Sci USA.

[49] Kim BB, Kim M, Park YH, Ko Y, Park JB (2017). Short-term application of dexamethasone on stem cells derived from human gingiva reduces the expression of RUNX2 and beta-catenin. J Int Med Res.

[50] Yuan JS, Reed A, Chen F, Stewart CN (2006). Statistical analysis of real-time PCR data. BMC Bioinformatics.

[51] Li Q, Kannan A, Das A, DeMayo FJ, Hornsby PJ, Young SL (2013). WNT4 Acts Downstream of BMP2 and Functions via β-Catenin Signaling Pathway to Regulate Human Endometrial Stromal Cell Differentiation. Endocrinology.

[52] Li S, Li N, Zhu P, Wang Y, Tian Y, Wang X (2015). Decreased β-catenin expression in first-trimester villi and decidua of patients with recurrent spontaneous abortion. J Obstet Gynaecol Res.

[53] Wang X, Zhang Z, Zeng X, Wang J, Zhang L, Song W (2018). Wnt/beta-catenin signaling pathway in severe preeclampsia. J Mol Histol.

[54] Li N, Li S, Wang Y, Wang J, Wang K, Liu X (2017). Decreased expression of WNT2 in villi of unexplained recurrent spontaneous abortion patients may cause trophoblast cell dysfunction via downregulated Wnt/β-catenin signaling pathway. Cell Biol Int.

[55] Hayashi K, Erikson DW, Tilford SA, Bany BM, Maclean JA, Rucker EB (2009). Wnt genes in the mouse uterus: potential regulation of implantation. Biol Reprod.

[56] Daikoku T, Song H, Guo Y, Riesewijk A, Mosselman S, Das SK (2004). Uterine Msx-1 and Wnt4 signaling becomes aberrant in mice with the loss of leukemia inhibitory factor or Hoxa-10: evidence for a novel cytokine-homeobox-Wnt signaling in implantation. Mol Endocrinol.

[57] Franco HL, Dai D, Lee KY, Rubel CA, Roop D, Boerboom D (2011). WNT4 is a key regulator of normal postnatal uterine development and progesterone signaling during embryo implantation and decidualization in the mouse. Faseb J.

[58] Nayeem SB, Dharmarajan A, Keelan JA (2015). Paracrine communication modulates production of Wnt antagonists and COX1-mediated prostaglandins in a decidual-trophoblast co-culture model. Mol Cell Endocrinol.

[59] Popovici RM, Betzler NK, Krause MS, Luo M, Jauckus J, Germeyer A (2006). Gene expression profiling of human endometrial-trophoblast interaction in a coculture model. Endocrinology.

[60] Freis A, Keller A, Ludwig N, Meese E, Jauckus J, Rehnitz J (2017). Altered miRNA-profile dependent on ART outcome in early pregnancy targets Wnt-pathway. Reproduction.

[61] Ring L, Neth P, Weber C, Steffens S, Faussner A (2014). beta-Catenin-dependent pathway activation by both promiscuous "canonical" WNT3a-, and specific "noncanonical" WNT4- and WNT5a-FZD receptor combinations with strong differences in LRP5 and LRP6 dependency. Cell Signal.

[62] Jeong JW, Lee HS, Franco HL, Broaddus RR, Taketo MM, Tsai SY (2009). beta-catenin mediates glandular formation and dysregulation of beta-catenin induces hyperplasia formation in the murine uterus. Oncogene.

[63] Messerschmidt D, de Vries WN, Lorthongpanich C, Balu S, Solter D, Knowles BB (2016). β-catenin-mediated adhesion is required for successful preimplantation mouse embryo development. Development.

[64] Patterson AL, Pirochta J, Tufano SY, Teixeira JM (2017). Gain-of-function β-catenin in the uterine mesenchyme leads to impaired implantation and decidualization. J Endocrinol.

[65] Lee KY, Jeong JW, Wang J, Ma L, Martin JF, Tsai SY (2007). Bmp2 is critical for the murine uterine decidual response. Mol Cell Biol.

[66] Li Q, Kannan A, Wang W, Demayo FJ, Taylor RN, Bagchi MK (2007). Bone morphogenetic protein 2 functions via a conserved signaling pathway involving Wnt4 to regulate uterine decidualization in the mouse and the human. J Biol Chem.

